# Cassiaside C Inhibits M1 Polarization of Macrophages by Downregulating Glycolysis

**DOI:** 10.3390/ijms23031696

**Published:** 2022-02-01

**Authors:** Ye Jin Kim, Sungwoo Lee, Jonghwa Jin, Hyein Woo, Yeon-Kyung Choi, Keun-Gyu Park

**Affiliations:** 1 Department of Internal Medicine, School of Medicine, Kyungpook National University, Kyungpook National University Hospital, Daegu 41944, Korea; freewilly59@hanmail.net (Y.J.K.); becauseofu77@gmail.com (J.J.); wooing86@gmail.com (H.W.); 2 Research Institute of Aging and Metabolism, Kyungpook National University, Daegu 41566, Korea; 3 New Drug Development Center, Daegu Gyeongbuk Medical Innovation Foundation, Daegu 41061, Korea; swlee@dgmif.re.kr; 4 Department of Internal Medicine, School of Medicine, Kyungpook National University, Kyungpook National University Chilgok Hospital, Daegu 41404, Korea

**Keywords:** M1 polarization, macrophage, glycolysis, Cassiaside C

## Abstract

Classically activated M1 macrophages reprogram their metabolism towards enhanced glycolysis to obtain energy and produce pro-inflammatory cytokines after activation by mammalian target of rapamycin complex 1 (mTORC1) and hypoxia-inducible factor (HIF)-1α. Thus, a strategy that constrains M1 polarization of macrophages via downregulation of glycolysis is essential for treating chronic inflammatory diseases. Cassiae semen has pharmacological activity against various inflammatory diseases. However, it is unclear whether specific compounds within Cassia seeds affect M1 polarization of macrophages. Here, we investigated whether Cassiaside C napthopyrone from Cassiae semen inhibits M1 polarization by downregulating glycolysis. We found that Cassiaside C reduced expression of inducible nitric oxide synthase and cyclooxygenase-2 and the phosphorylation of nuclear factor kappa B, all of which are upregulated in lipopolysaccharide (LPS)/interferon (IFN)-γ-treated Raw264.7 cells and peritoneal macrophages. Moreover, Cassiaside C-treated macrophages showed marked suppression of LPS/IFN-γ-induced HIF-1α, pyruvate dehydrogenase kinase 1, and lactate dehydrogenase A expression, along with downregulation of the phosphoinositide 3-kinases (PI3K)/AKT/mTORC1 signaling pathway. Consequently, Cassiaside C attenuated enhanced glycolysis and lactate production, but rescued diminished oxidative phosphorylation, in M1 polarized macrophages. Thus, Cassiaside C dampens M1 polarization of macrophages by downregulating glycolysis, which could be exploited as a therapeutic strategy for chronic inflammatory conditions.

## 1. Introduction

Glycolysis provides cells with a very rapid supply of energy to support biosynthesis; thus, increased glycolysis is a key feature of proliferating cells, including cancer cells, vascular smooth muscles cells, and immune cells [1,2,3]. Immune cells, such as neutrophils, macrophages, and T lymphocytes, also rely heavily on glycolysis to generate cellular adenosine triphosphate (ATP) and biosynthetic precursors and for redox buffering capacity, all of which ensure adequate function and development [4,5,6,7]. Accumulating evidence shows that macrophages possess distinct metabolic reprogramming pathways that correlate with their functional status [8]. In general, M1 macrophages express inducible nitric oxide synthase (iNOS), which generates nitric oxide (NO) from arginine, thereby reprogramming their metabolism towards enhanced glycolysis to drive upregulated cytokine production in response to inflammatory signals [9].

Mammalian target of rapamycin complex 1 (mTORC1) is a master cell growth factor that acts as a crucial regulator of cellular energy metabolism [10]. mTORC1 is associated with the activation of glycolysis via increased translation or transcription of glycolytic enzymes [11] and has a direct effect on macrophage polarization [12]. Hypoxia-inducible factor (HIF)-1α, which is regulated by mTORC1, participates in regulation of M1 polarization by accelerating expression of glycolysis-related genes GLUT1, HK, and LDHA [13]. Moreover, the phosphoinositide 3-kinases (PI3K)/AKT signaling pathway mediates the effector responses of macrophages through regulation of mTORC1, thereby regulating innate immune responses [14]. Thus, manipulating mTORC1 activity and macrophage polarization has therapeutic potential for chronic inflammatory disease [15].

Cassiae semen, a well-known Asian herbal medicine derived from the dried seeds of *Cassia obtusifolia* L., has therapeutic activity against Alzheimer’s disease, cerebral global ischemia, acute liver injury, inflammation, and hypertension [16,17]. Among more than 70 compounds in Cassiae semen, anthraquinone and naphthopyrone are the most structurally diverse and biologically active [18]. Previous reports have focused on the diverse pharmacological activities (antihyperlipidemic, antidiabetic, and neuroprotective) of anthraquinones [16,19,20]. These pharmacological effects are mediated via the PI3K/AKT and nuclear factor kappa B (NF-κB) signaling pathways [21,22]. However, the pharmacological activity of napthopyrones from Cassiae semen is unknown.

Here, we investigated whether napthopyrone from Cassiaside C inhibits glycolysis by suppressing PI3K/AKT/mTORC1 signaling, thereby inhibiting lipopolysaccharide (LPS)/interferon (IFN)-γ-induced M1 polarization of macrophages.

## 2. Results

### 2.1. Cassiaside C Inhibits LPS/IFN-γ-Induced M1 Polarization of Macrophages

**Figure 1:**
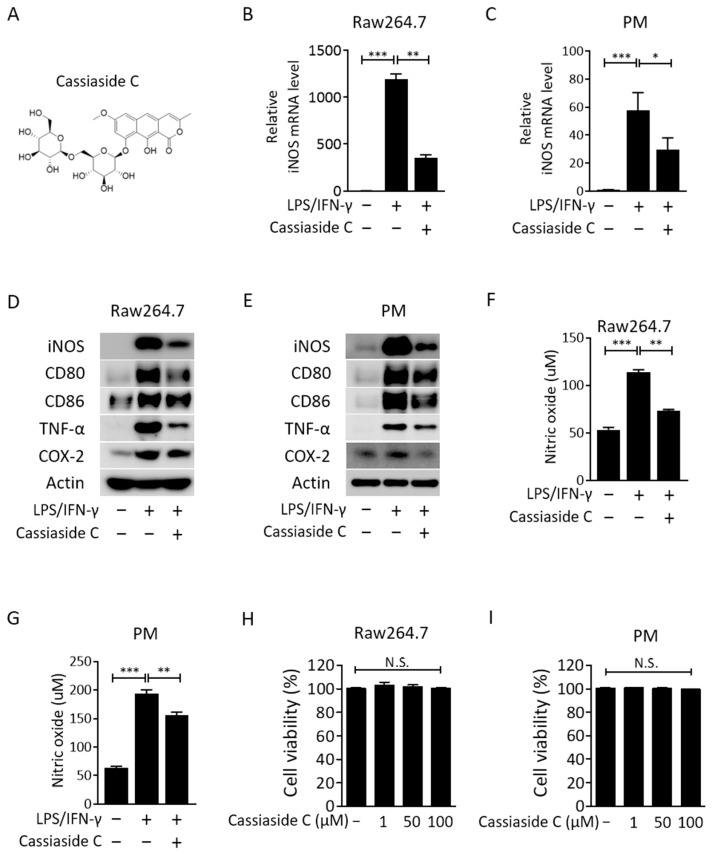
Cassiaside C inhibits M1 macrophage polarization. (**A**) Chemical structure of Cassiaside C. (**B**,**C**) Effects of Cassiaside C on *iNOS* mRNA expressions in LPS/IFN-γ-stimulated Raw264.7 cells and PMs. (**D**,**E**) Effects of Cassiaside C on levels of iNOS, CD80, CD86, TNF-α, and COX-2 in LPS/IFN-γ-stimulated Raw264.7 cells and PMs. (**F**,**G**) Effects of Cassiaside C on NO concentrations in LPS/IFN-γ-stimulated Raw264.7 cells and PMs. (**H**,**I**) Effects of Cassiaside C on cellular viability in LPS/IFN-γ-stimulated Raw264.7 and PM cells. Data are expressed as the mean ± SEM (*n* = 3 technical replicates). N.S., not significant; * *p* < 0.05, ** *p* < 0.01, and *** *p* < 0.001. PM, peritoneal macrophages.

The chemical structure of Cassiaside C is shown in Figure 1A. The effects of Cassiaside C on M1 polarization were performed using the popular murine macrophage cell line Raw264.7 and peritoneal macrophages. Raw264.7 cells, derived from BALB/c mice infected with Abelson leukemia virus, are one of most commonly used cell lines for in vitro and in vivo studies of monocyte/macrophages [23]. Treatment of Raw264.7 cells and peritoneal macrophages with LPS/IFN-γ increased expression of *iNOS* mRNA and protein, both of which were attenuated significantly by treatment with Cassiaside C (Figure 1B–E). Moreover, LPS/IFN-γ-induced upregulation of M1 phenotype markers, such as CD86, CD80, and TNF-α, was effectively attenuated by treatment with Cassiaside C (Figure 1D,E). Treatment with Cassiaside C markedly reduced LPS/IFN-γ-induced production of NO in Raw264.7 cells and peritoneal macrophages (Figure 1F,G). In addition, expression of cyclooxygenease-2 (COX-2; another M1 marker), mRNA, and protein was upregulated in response to LPS/IFN-γ; again, this was attenuated by treatment with Cassiaside C (Figure 1D,E). A cell viability assay showed that Cassiaside C was not cytotoxic at concentrations 50–100 μM (Figure 1H,I). Taken together, these data indicate that Cassiaside C inhibits LPS/IFN-γ-induced M1 polarization of macrophages.

### 2.2. Cassiaside C Inhibits Pro-Inflammatory Cytokine Expression and Secretion by LPS/IFN-γ-Induced M1 Macrophages

**Figure 2:**
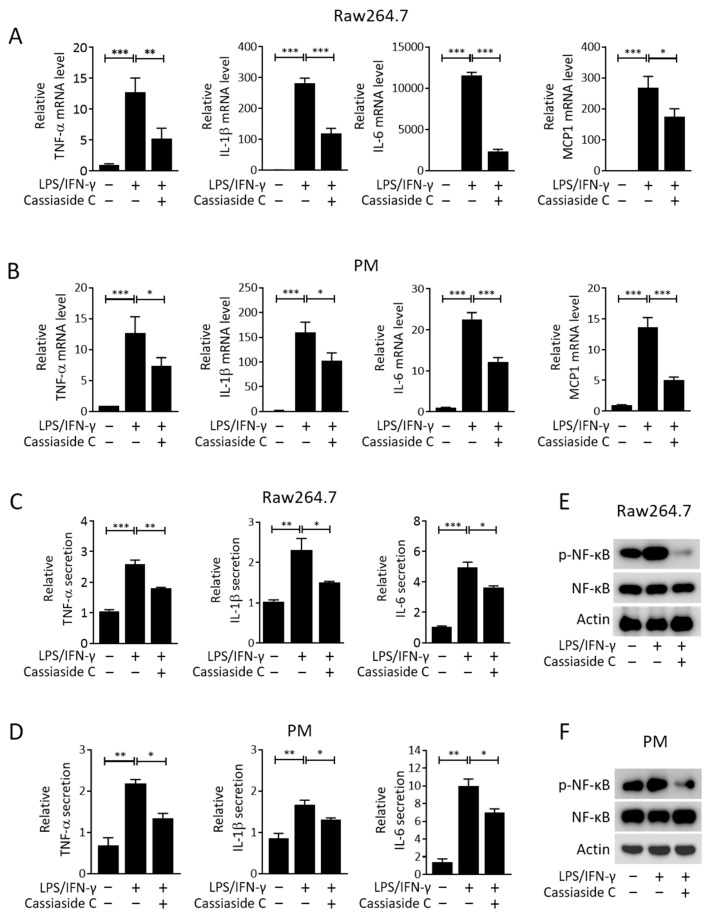
Cassiaside C suppresses LPS/IFN-γ-stimulated pro-inflammatory cytokine expression and secretion by macrophages. (**A**,**B**) Effects of Cassiaside C on *TNF-α, IL-β, IL-6,* and *MCP-1* mRNA expression in LPS/IFN-γ-stimulated Raw264.7 cells and PMs. (**C**,**D**) Effects of Cassiaside C on secretion of TNF-α, IL-β, and IL-6 in LPS/IFN-γ-stimulated Raw264.7 cells and PMs. (**E**,**F**) Effects of Cassiaside C on phosphorylated NF-κB in LPS/IFN-γ-stimulated Raw264.7 cells and PMs. Data are expressed as the mean ± SEM (*n* = 3 technical replicates). * *p* < 0.05, ** *p* < 0.01, and *** *p* < 0.001. PM, peritoneal macrophages.

Consistent with previous reports showing that inhibition of M1 polarization reduces production of cytokines [24,25,26], we observed that production of mRNA encoding pro-inflammatory cytokines tumor necrosis factor (TNF-α), interleukin (IL)-1β, and IL-6, as well as chemokines monocyte chemotactic protein (MCP)-1, by Raw264.7 cells and peritoneal macrophages in response to LPS/IFN-γ was inhibited strongly by Cassiaside C (Figure 2A,B). Moreover, Cassiaside C also inhibited secretion of TNF-α, IL-1β, and IL-6 by LPS/IFN-γ-stimulated macrophages (Figure 2C,D). Phosphorylation of NF-κB, a major transcription factor required for expression of pro-inflammatory factors [10], was upregulated in Raw264.7 cells and peritoneal macrophages treated with LPS/IFN-γ; however, this was attenuated by Cassiaside C (Figure 2E,F). Overall, the data show that LPS/IFN-γ-induced inflammatory reactions, accompanied by an increase in iNOS and inflammatory cytokines, are effectively inhibited by Cassiaside C, leading to inhibition of M1 polarization.

### 2.3. Cassiaside C Inhibits the PI3K/AKT/mTORC1 Signaling and Glycolytic Pathways in LPS/IFN-γ-Induced M1 Macrophages

**Figure 3:**
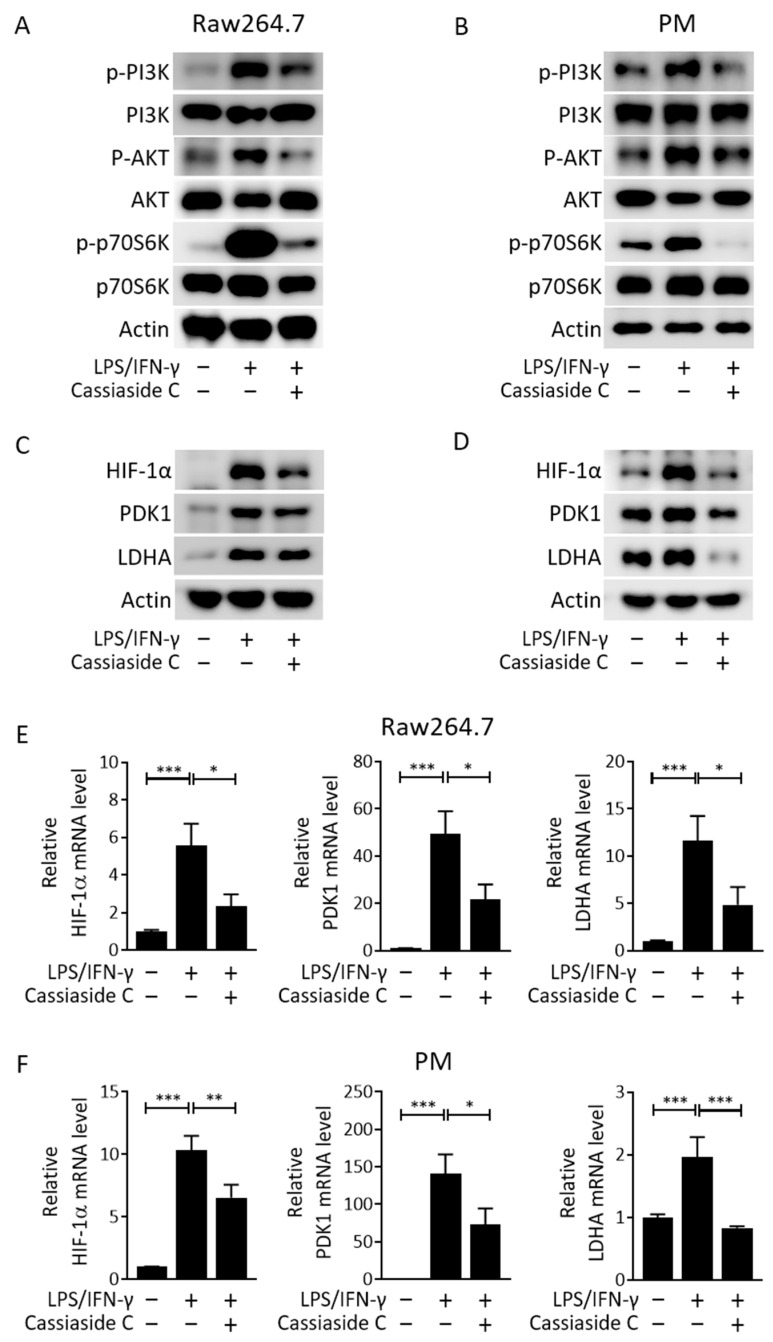
Cassiaside C attenuates LPS/IFN-γ-stimulated mTORC1 activity and the glycolytic pathway. (**A**,**B**) Effects of Cassiaside C on phosphorylated PI3K, AKT, and p70S6K in LPS/IFN-γ-stimulated Raw264.7 cells and PMs. (**C**–**F**) Effects of Cassiaside C on HIF-1α, PDK1, and LDHA protein levels (**C**,**D**) and mRNA expression (**E**,**F**) in LPS/IFN-γ-stimulated Raw264.7 cells and PMs. Data are expressed as the mean ± SEM (*n* = 3 technical replicates). * *p* < 0.05, ** *p* < 0.01, and *** *p* < 0.001. PM, peritoneal macrophages.

Given that the PI3K/AKT/mTORC1 signaling pathway is involved in LPS/IFN-γ-stimulated M1 polarization [11,27], we investigated the effects of Cassiaside C on this signaling pathway in the context of M1 polarization. In agreement with previous results [27,28], we found that treatment with LPS/IFN-γ increased phosphorylation of PI3K (Y458), AKT (T308), and S6 kinase (S6K; T389) in Raw264.7 cells and peritoneal macrophages and that this was alleviated by treatment with Cassiaside C (Figure 3A,B). The PI3K/AKT/mTOR pathway increases production of hypoxia-inducible factor (HIF)-1α, the overexpression of which upregulates expression of glycolytic genes such as pyruvate dehydrogenase kinase 1 (PDK1) and lactate dehydrogenase A (LDHA) [29]; therefore, we next investigated whether Cassiaside C inhibits expression of HIF-1α, PDK1, and LDHA in LPS/IFN-γ-stimulated M1 macrophages. The results showed that Cassiaside C treatment reduced the levels of HIF-1α, PDK1, and LDHA mRNA and protein in LPS/IFN-γ-treated Raw264.7 cells and peritoneal macrophages (Figure 3C–F).

### 2.4. Cassiaside C Inhibits Glycolysis and Lactate Production by LPS/IFN-γ-Induced M1 Macrophages

**Figure 4:**
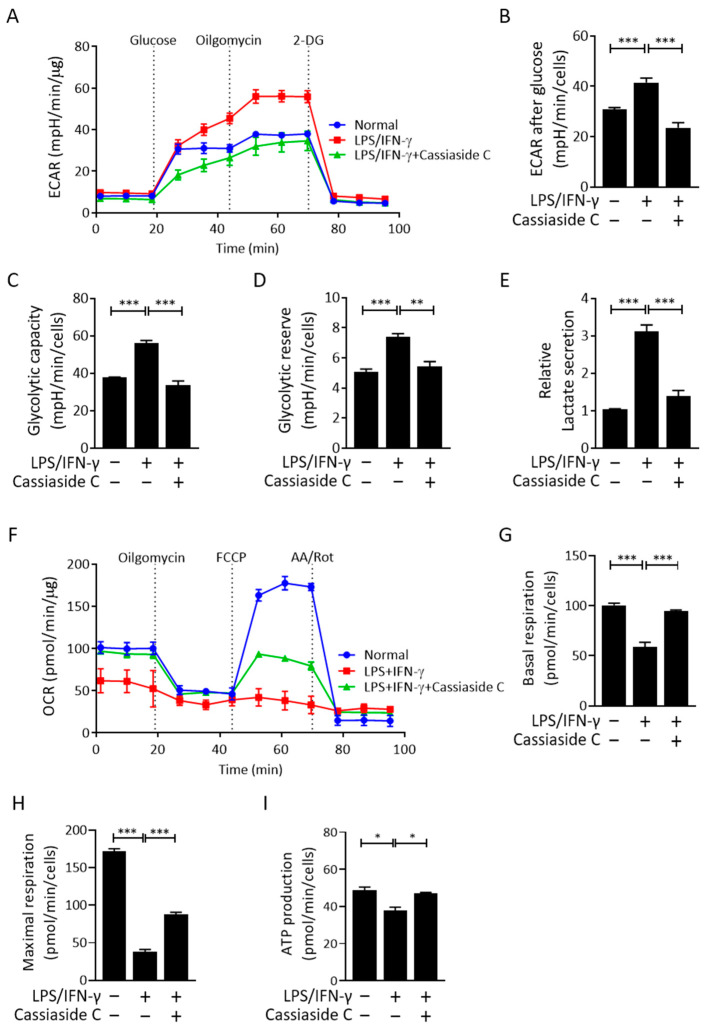
Cassiaside C suppresses glycolysis but increases mitochondrial respiration in LPS/IFN-γ-stimulated macrophages. (**A**–**E**) The ECAR in LPS/IFN-γ-stimulated macrophages. ECAR kinetic traces (**A**,**B**), glycolytic capacity and reserve (**C**,**D**), and lactate levels (**E**) in LPS/IFN-γ-stimulated macrophages treated with or without Cassiaside C. (**F**–**I**) OCR kinetic traces (**F**) and rates of basal (**G**), maximal (**H**), and ATP-linked (**I**) respiration in LPS/IFN-γ-stimulated macrophages treated with or without Cassiaside C. Data are expressed as the mean ± SEM (*n* = 3 technical replicates). * *p* < 0.05, ** *p* < 0.01, and *** *p* < 0.001. 2-DG, 2-deoxyglucose 2-Dexyo glucose. AA, antimycin A/Rot, rotenone.

The inhibitory effects of Cassiaside C on glycolytic signaling and gene expression led us to further investigate whether Cassiaside C inhibits glycolysis and lactate production in LPS/IFN-γ-induced M1 macrophages. Consistent with the downregulation of HIF-1α, PDK1, and LDHA, we found that Cassiaside C suppressed glycolysis and the glycolytic capacity of LPS/IFN-γ-stimulated peritoneal macrophages, as assessed by measuring the extracellular acidification rate (ECAR) (Figure 4A–D). LPS/IFN-γ-induced lactate production was also abolished by treatment with Cassiaside C (Figure 4E). In contrast to the ECAR, Cassiaside C rescued the decrease in basal and maximal oxygen consumption rate (OCR) in LPS/IFN-γ-treated macrophages (Figure 4F–H).

## 3. Discussion

Here, we found that Cassiaside C inhibited M1 polarization of macrophages in response to LPS/IFN-γ stimulation by downregulating glycolysis. The results suggest that the anti-inflammatory efficacy of Cassiaside C is attributable to suppression of PI3K/AKT/mTORC1 signaling in LPS/IFN-γ-stimulated macrophages.

Macrophages can be broadly classified as M1 or M2, although this is somewhat simplistic because there is a more complicated relationship between these phenotypes [30]. Inflammatory macrophages trigger a metabolic switch that induces Warburg-like upregulation of aerobic glycolysis while, at the same time, impairing oxidative phosphorylation, thereby exacerbating inflammation [31,32]. A previous report demonstrated that obesity-associated metabolic stress in macrophages provokes metabolic reprogramming towards glycolysis; therefore, targeting glycolysis is a possible therapeutic option for chronic inflammatory diseases [33]. In the present study, we showed that Cassiaside C, the main bioactive compound in Cassiae semen, reduced expression of iNOS and pro-inflammatory cytokines in LPS/IFN-γ-stimulated macrophages. Consistent with previous reports showing that pharmacological inhibition of glycolysis inhibits M1 polarization [34,35], we observed that Cassiaside C abrogated LPS/IFN-γ-induced glycolysis, as evidenced by a reduced ECAR and suppression of genes implicated in the glycolytic program (i.e., PDK1 and LDHA). Considering that LPS/IFN-γ blunts mitochondrial oxidative respiration in macrophages, Cassiaside C-induced restoration of mitochondrial function may prevent repolarization of macrophages towards a pro-inflammatory phenotype [36].

Accumulating evidence suggests different metabolic routes that regulate glycolysis in rapidly proliferating cells [37,38]. In macrophages, LPS signaling-induced AKT activation increases glucose uptake and aerobic glycolysis to support inflammatory cytokine production and a respiratory burst [39]. Studies show that the AKT/mTORC1 pathway potentiates the glycolytic program by translating HIF-1α mRNA or by stabilizing HIF-1α expression which, in turn, activates transcription of glycolytic genes [40,41]. Moreover, M1-polarized macrophages induce HIF-1α expression, and HIF-1α deficiency in macrophages, resulting in defects in key cellular functions, including motility, aggregation, and invasion [42,43]. Consistent with previous studies showing that mTORC1 and HIF-1α mediate glycolysis in response to LPS signaling, we showed that both the PI3K/AKT/mTORC1 pathway and HIF-1α are implicated in Cassiaside C-mediated inhibition of M1 polarization which, in turn, downregulates transcription of PDK1 and LDHA. Inducible expression of pro-inflammatory cytokines is regulated by AKT/mTORC1 signaling but also directly by NF-κB [44]. When combined with mutual regulation of AKT/mTORC1 and NF-κB signaling [45,46,47], it may explain the non-concordance between cytokine production and phosphorylated NF-kB levels observed in our results. Further studies should investigate how Cassiaside C abrogates PI3K/AKT/mTORC1 and NF-κB signaling.

In summary, we show here that Cassiaside C abrogates M1 polarization of macrophages by inhibiting PI3K/AKT/mTORC1 signaling and glycolysis. Thus, Cassiaside C is a potential treatment for M1 macrophage-mediated inflammatory diseases.

## 4. Materials and Methods

### 4.1. Isolation of Peritoneal Macrophages

Peritoneal macrophages were isolated from mice, as previously described [48]. Briefly, 8-week-old C57BL/6J mice were injected intraperitoneally (i.p.) with 3% thioglycollate broth and then sacrificed 4 days later. After injection of 5 mL of cold PBS into the peritoneum, followed by gentle massage, the peritoneal fluid was drained and dispensed into a 50 mL conical polypropylene centrifuge tube on ice. The peritoneal exudate cells were pelleted by centrifugation for 10 min at 500× *g* in a refrigerated (4 °C) centrifuge. Peritoneal macrophages were seeded in 96-well plates at a density of 3 × 10^6^ cells per well. All animal procedures were approved by the Institutional Animal Care and Use Committee (IACUC) of Kyungpook National University (KNU2021-0191, approved in 10 May 2021).

### 4.2. Culture of Raw264.7 Cells

Raw264.7 cells (murine monocyte/macrophage-like cells) were purchased from Korean Cell Line Bank (KCLB, Seoul, Korea) and cultured at 37 °C/5% CO_2_ in high-glucose DMEM containing 10% FBS, 1% penicillin, and streptomycin. For all experiments, cells were grown to between 80–90% confluence and passaged no more than ten times.

### 4.3. Macrophage Differentiation

Peritoneal macrophages and Raw264.7 cells were plated for 24 h before the experiments. The cells were then stimulated for 18–24 h with 100 ng/mL LPS (Sigma-Aldrich Co., St. Louis, MO, USA) and 10 ng/mL IFN-γ (R&D systems Inc., St. Paul, MN, USA) to induce polarization to an M1 phenotype.

### 4.4. Chemical Treatment

Cassiaside C (M.W, 569.5 g, Purity ≥ 98%) was purchased from ChemFaces (Wuhan, China).

### 4.5. Cell Viability Assay

Cell viability was measured in a CCK-8 assay. Briefly, Raw264.7 cells (1 × 10^4^) and peritoneal macrophages (3 × 10^4^) were seeded in 96-well plates and incubated overnight at 37 °C. Cells were then treated with Cassiaside C (10–100 μM) for 24 h. Finally, 10 μL of CCK-8 was added to each to yield a final concentration of 0.5 mg/mL, and ODs were analyzed by using a Tecan Spark ELISA reader at 450 nm.

### 4.6. Western Blot Analysis

Protein expression was determined by Western blotting, which was performed according to the method of Lee et al. [49]. Briefly, proteins from cellular lysates were separated on SDS-PAGE gels and transferred to PVDF membranes (Millipore, Danvers, MA, USA). After blocking for 1 h with 5% skim milk in TBST buffer, the membranes were incubated overnight at 4 °C with antibodies specific for the following proteins: iNOS (BD Biosciences, San Jose, CA, USA), COX-2 (Santa Cruz Biotechnology, Santa Cruz, CA, USA), CD80, CD86, TNF-α, phosphor-PI3K p85 (Tyr458), PI3K, phosphor-AKT (T308), AKT, phospho-p70S6K (T389), p70S6K, phosphor-NF-κB (Ser536), NF-κB, PDK1, LDHA (Cell Signaling Technology; Danvers, MA, USA), HIF1-α (Novus Biologicals, Building IV Centennial, CO, USA), and β-actin (Sigma). After three washes in TBST, membranes were incubated with HRP-conjugated secondary antibodies (GeneTex, Irvine, CA, USA). HRP was detected using the ECL reagent (BioNote, Hwaseong, Gyeonggi-do, Korea).

### 4.7. Real-Time PCR

Total RNA was harvested using TRIzol reagent (Qiagen, Hilden, Germany). RNA was quantified using a NanoDrop spectrophotometer (Thermo Fisher Scientific, Waltham, MA, USA). Then, complementary DNA (cDNA) was synthesized using the RevertAid First Strand cDNA Synthesis Kit (Thermo Fisher Scientific, Waltham, MA, USA). SYBR green PCR Master Mix (Applied Biosystems, Foster City, CA, USA) and a VIIA 7 Real-time PCR system instrument (Applied Biosystems) were used for analysis. The cycling parameters were as follows: 95 °C for 2 min, followed by 40 cycles at 95 °C for 5 s and 60 °C for 30 s. Gene expression was normalized against the corresponding level of GAPDH mRNA. The primer sequences are shown in Table 1.

### 4.8. Measurement of the OCR and ECAR

The ECAR and OCR of peritoneal macrophages were analyzed using an XF-24 Extracellular Flux Analyzer (Seahorse Bioscience, North Billerica, MA, USA). First, peritoneal macrophages were seeded into a Seahorse XF24 plate. After 24 h, 100 μM Cassiaside C was added with or without 100 ng/mL LPS and 100 ng/mL IFN-γ for an additional 18 h. Glucose (10 mM; Sigma), oligomycin (1 μM; Sigma), and 2-deoxyglucose (100 mM; Sigma) were added at the indicated time points during ECAR measurement. Oligomycin (10 μM; Sigma), carbonyl cyanide 3-chlorophenylhydrazone (FCCP, 50 μM; Sigma), and antimycin A/rotenone (10 μM/50 μM Sigma) were added at the indicated times during OCR measurement.

### 4.9. Cytokine Measurement

To measure cytokine production, peritoneal macrophages (3 × 10^5^ cells/well) and Raw264.7 cells (1 × 10^5^ cells/well) were plated in a 24-well plate and stimulated with LPS/IFN-γ with or without Cassiaside C for 18–24 h. Then, mouse TNF-α, IL-β, and IL-6 ELISA kits (Thermo Fisher Scientific) were used to measure mouse cytokines in the collected culture medium. Data were normalized against the protein concentration at each cell harvest.

### 4.10. NO Assay

For NO analysis, peritoneal macrophages (3 × 10^4^ cells/well) and Raw264.7 (1 × 10^4^) macrophages were seeded in 96-well plates and stimulated with LPS/IFN-γ with or without Cassiaside C for 18–24 h. NO in the cell culture medium was measured using the Griess assay (Promega Co., Madison, WI, USA). Each 100 μL of cell culture medium was mixed with 100 μL of Griess reagent and incubated at room temperature for 10 min, and the absorbance at 540 nm was read using microplate reader. Data were normalized to the cell number.

### 4.11. Lactate Measurement

Lactate concentrations in culture medium were measured using the Lactate-Glo assay (Promega, J5021). Peritoneal macrophages (3 × 10^4^ cells/well) and Raw264.7 (1 × 10^4^) macrophages were seeded in 96-well plates and stimulated with LPS/IFN-γ with or without Cassiaside C for 18–24 h. Data were normalized to the cell number.

### 4.12. Statistical Analysis

All data are presented as the mean ± SEM and are representative of at least three independent experiments. Statistical analyses were performed using GraphPad 7 software. Data were compared using Student’s *t*-test.

## Figures and Tables

**Table 1:** List of real-time PCR primers.

Gene	Forward	Reverse
*iNOS*	GGCAGCCTGTGAGACCTTTG	TGCATTGGAAGTGAAGCGTTT
*TNF-* *α*	TTCTCATTCCTGCTTGTGGC	CTGATGAGAGGGAGGCCATT
*IL-1* *β*	CTTTCCCGTGGACCTTCCAG	AATGGGAACGTCACACACCA
*IL-6*	TTGCCTTCTTGGGACTGATG	CTCATTTCCACGATTTCCCA
*MCP-1*	TGTGCTGACCCCAAGAAGGA	GTGCTTGAGGTGGTTGTGGA
*HIF-1*	TCAAGTCAGCAACGTGGAAG	TATCGAGGCTGTGTCGACTG
*PDK1*	TTACGGATTGCCCATATCACG	CCCGGTCACTCATCTTCACAGT
*LDHA*	CAAAGTCCAAGATGGCAACCC	AGCACCAACCCCAACAACTGT
*GAPDH*	GCTGACAATCTTGAGTGAGT	GAAGGGTGGAGCCAAAAG

*iNOS*, inducible nitric oxide synthase; *TNF-α*, tumor necrosis factor-α; *IL-1β*, interlukin-1β; *IL-6*, interlukin-6; *MCP-1*, monocyte chemotactic protein-1; *HIF-1α*, hypoxia-inducible factor-1α; *PDK1*, pyruvate dehydrogenase kinase 1; *LDHA*, lactate dehydrogenase A; *GAPDH*, housekeeping gene.

## Data Availability

The data presented in this study are available on request from the corresponding author.
